# Limited effects of intravenous paracetamol on patent ductus arteriosus in very low birth weight infants with contraindications for ibuprofen or after ibuprofen failure

**DOI:** 10.1007/s00431-015-2541-5

**Published:** 2015-04-30

**Authors:** Daniëlla W. E. Roofthooft, Ingrid M. van Beynum, Johan C. A. de Klerk, Monique van Dijk, John N. van den Anker, Irwin K. M. Reiss, Dick Tibboel, Sinno H. P. Simons

**Affiliations:** Department of Pediatrics, Division of Neonatology, Erasmus MC—Sophia Children’s Hospital, Room Sp2463, Wytemaweg 80, 3015 CN Rotterdam, The Netherlands; Department of Pediatric Cardiology, Erasmus MC—Sophia Children’s Hospital, Rotterdam, The Netherlands; Intensive Care and Department of Pediatric Surgery, Erasmus MC—Sophia Children’s Hospital, Rotterdam, The Netherlands; Department of Pediatric Pharmacology, University Children’s Hospital, Basel, Switzerland

**Keywords:** Paracetamol, Patent ductus arteriosus, VLBW infants, Ibuprofen contraindication, Ibuprofen treatment failure

## Abstract

Finding the optimal pharmacological treatment of a patent ductus arteriosus (PDA) in preterm neonates remains challenging. There is a growing interest in paracetamol as a new drug for PDA closure. In this prospective observational cohort study, we evaluated the effectiveness of intravenous paracetamol in closing a PDA in very low birth weight infants with a hemodynamically significant PDA who either did not respond to ibuprofen or had a contraindication for ibuprofen. They received high-dose paracetamol therapy (15 mg/kg/6 h intravenous) for 3–7 days. Cardiac ultrasounds were performed before and 3 and 7 days after treatment. Thirty-three patients were included with a median gestational age of 25^1/7^ weeks (IQR 1.66), a median birth weight of 750 g (IQR 327), and a median postnatal age of 14 days (IQR 12). Paracetamol was ineffective in 27/33 patients (82 %). Even more, after previous exposure to ibuprofen, this was even 100 %.

*Conclusion*: In this study, paracetamol after ibuprofen treatment failure was not effective for PDA closure in VLBW infants. From the findings of this study, paracetamol treatment for PDA closure cannot be recommended for infants with a postnatal age >2 weeks. Earlier treatment with paracetamol for PDA might be more effective.

**Table:** 

**What is known:** • *The ductus arteriosus fails to close after birth in 30 to 60* % *of prematurely born neonates and is a significant cause of morbidity and mortality in these infants.* • *Paracetamol gained importance as an alternative drug in PDA closure.*
**What is new:** • *Paracetamol for PDA closure after ibuprofen treatment failure was not effective in VLBW infants.* • *Effect of paracetamol on PDA closure was observed when given as primary treatment.*

## Introduction

The ductus arteriosus fails to close after birth in 30 to 60 % of prematurely born infants [16]. This condition—patent ductus arteriosus (PDA)—is associated with a prolonged ventilation need and carries an increased risk of morbidity (i.e., necrotizing enterocolitis, chronic lung disease) and even mortality [10, 15, 18]. Pharmacological closure with non-steroidal anti-inflammatory drugs (NSAIDs), mainly ibuprofen and indomethacin, is currently the standard of care [27]. NSAIDs are not effective in around 30 % of patients, however, and can have side effects such as gastrointestinal bleeding and perforation, diminished platelet aggregation, hyperbilirubinemia, and transient renal function impairment [22, 23]. Moreover, NSAIDs are contraindicated in a considerable proportion of newborns, notably those with renal failure, intracerebral hemorrhage, gastrointestinal problems, and thrombocytopenia. If NSAIDs fail or are contraindicated, the only currently available solution is surgical ligation, which is associated with the risks of cardiothoracic surgery and impaired neurological outcome [17]. Therefore, alternative pharmacological interventions are needed.

Paracetamol has been suggested as an alternative drug for PDA closure [21]. More than 10 observational and retrospective studies have described oral or intravenous high-dose paracetamol treatment with varying effectiveness [1, 8, 9, 11, 12, 19, 21, 26, 29] (see Table 1). Two recent prospective randomized controlled trials comparing oral paracetamol with ibuprofen both showed a slightly favorable effect of paracetamol (closure rate 81.2 versus 78.8 % for ibuprofen) [5]. The other trial by Oncel et al. even showed a higher closure success rate in the paracetamol group (97.5 versus 95 % in the ibuprofen group) after a maximum of two courses of ibuprofen or paracetamol [20].

**Table 1:** Literature review on PDA treatment with paracetamol

Author	Dose (mg/kg/day)	Treatment interval (h)	Route	Treatment PCM (days)	Gestational age (weeks)	PN age start PCM (days)	Patients that achieved ductal closure/no surgical ligation
1. Hammerman 2011	15	6	Oral	Max. 7	26–29^6/7^	3–35	5/5
2. Yurttutan 2013	15	6	Oral	Max. 6	26–30	3–7	5/6
3. Oncel 2013	15	6	Intravenous	3–6	24–29	2–15	10/10
4. Alan 2013	15	6	Intravenous	Max. 19	26^2/7^–33^5/7^	8–19	0/3
5. Özmert 2013	15	6	Oral	3–6	23–32	20–47	5/7
6. Sinha 2013	15	8	Oral	2	27–33	4–7	10/10
7. Kessel 2013	15	6	Oral	3–11	26–30	?	7/7
8. Jasani 2013	15	6	Oral	2.3–4.3	28.5–31.1		2.6–8.9
9. Dang 2013 RCT	15	6	Oral	3	31.2 ± 1.8		65/80
10. Oncel 2013 RCT	15	6	Oral	3–6	≤26	2–3	12/13
	15	6	Oral	3–6	<28	2–3	22/23
11. Tekgunduz 2014	15	6	Intravenous	1	29	3	0/1^a^
	10	8	Intravenous	1–4	24–31	2–9	10/12^b^
12. Nadir 2014	15	6	Oral	Max. 7	24–27	2–22	4/7
13. el-Khuffash 2014	15	6	Oral	2	26–33	14–56	0/5
	15	6	Oral	7	26–30	8–35	6/7
	15	6	Intravenous	2–5	26–32	3–41	9/9
14. Terrin 2014	7.5–15 max. 60	4–6	Intravenous	3	26 ± 2	2.8 ± 1.2	6/8
15. Roofthooft 2014	15	6	Intravenous	3–7.5	23^6/7^–26^4/7^	3–33	6/33
16. el-Khuffash 2015	15	6	Intravenous	Max. 6	24.6–27.9	16–39	24/30

^a^Transaminases elevated: paracetamol treatment stopped after three doses, ductal closure with oral ibuprofen

^b^Ductal closure of two remaining PDAs with oral ibuprofen after paracetamol

After publication of the first studies on paracetamol and PDA closure, we added intravenous paracetamol to our PDA treatment guideline. However, the results in the first patients did not match the high closure rates of other studies, and only 20 % of patients did not need further PDA treatment after paracetamol [24]. Based on the promising results of other published studies, we decided to continue paracetamol treatment for PDA closure in preterm infants with ibuprofen contraindications or ibuprofen treatment failure.

In the current study, we describe the evaluation of the efficacy of intravenous paracetamol on PDA closure in very low birth weight (VLBW) infants admitted to our hospital.

## Methods

In a prospective observational, single center study performed from December 2012 until September 2014, at the level III NICU of the Erasmus MC—Sophia Children’s Hospital in Rotterdam, the Netherlands, we included all neonates with a gestational age of less than 28 weeks and a birth weight of less than 1500 g, diagnosed with a hemodynamically significant patent ductus arteriosus (hsPDA) using clinical and cardiac ultrasound evaluation. Findings in the first nine patients in the current study were also presented in a preliminary report in 2014 [4].

The first drug of choice in our department for PDA treatment was intravenous ibuprofen (Neobrufen^©^; Pedea ©), a single daily dose for 3 days (10 mg/kg on the first day, 5 mg/kg on the second and third days), and a repeated 3-day course if closure was not yet obtained. Paracetamol (Perfalgan©; Bristol-Meyers Squibb) was given if two courses of ibuprofen had no effect or if ibuprofen was contraindicated. Intravenous paracetamol 15 mg/kg every 6 h (60 mg/kg/day) was administered for a minimum of 3 days and a maximum of 7 days.

**Fig. 1:**
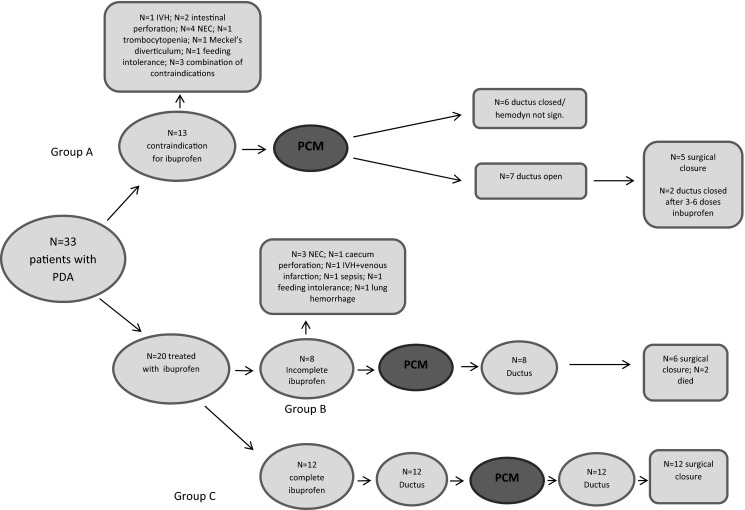
Flowchart included patients

Based on the indication for paracetamol, we created three groups. Group A: ibuprofen contraindicated and paracetamol as first drug of choice (primary contraindication); group B: development of a contraindication for ibuprofen during treatment with ibuprofen as first choice and switch to paracetamol (incomplete ibuprofen); and group C: two complete courses of ibuprofen failed to achieve closure and switch to paracetamol (complete ibuprofen) (see Fig. 1).

Contraindications for ibuprofen treatment were active intracerebral hemorrhage, thrombocytopenia or other known clotting disorders, severe sepsis, suspected or confirmed necrotizing enterocolitis (NEC), intestinal perforation, liver and kidney function disorders (oliguria <1.0 ml/kg/h, serum creatinine >110 μmol/l) and severe hyperbilirubinemia.

Echocardiographic examination was performed by the echocardiosonographer or pediatric cardiologist before the start of paracetamol treatment, after 3 days and after 7 days. Measurements included ductus diameter, PDA diameter/LPA (left pulmonary artery) diameter, and LA/Ao (left atrial to aortic root) ratio. Two-dimensional color Doppler echocardiography was performed using a Vivid-S6 (GE Health Care) ultrasound system equipped with a 10-MHz transducer.

Cardiac ultrasound studies were done at the bedside by different echocardiosonographers, and measurements were checked by one pediatric cardiologist (I. M. v B.). HsPDA closure was defined as no flow through the duct. An open duct with diameter <1.5 mm, without significant left-to-right shunt, PDA:LPA diameter <0.5 and LA/Ao ratio <1.4 was defined as small PDA and not hemodynamically significant. HsPDA was defined as a ductal diameter of >2.0 mm, PDA:LPA diameter >0.8 and/or LA/Ao ratio >1.6.

## Statistical analysis

Patient characteristics are presented as medians (IQR: interquartile range) in case of non-normally distributed variables and as means (standard deviations) in case of normally distributed variables. PDA diameters, PDA:LPA ratio and LA/Ao ratio before, during, and after treatment with paracetamol were compared using paired *t* tests. Fisher exact tests were used in case of categorical variables. Data analyses were performed with SPSS version 21.0 (SPSS Inc.). A *p* value of 0.05 was set as statistically significant.

## Results

A total of 33 VLBW infants with a median gestational age of 25^1/7^ weeks (range 23^6/7^–26^6/7^, IQR 1.66) and a median birth weight of 750 g (range 365–1130, IQR 327) were included. Intravenous paracetamol was started at a median postnatal age of 14 days (IQR 12). Background characteristics and clinical outcome are shown in Table 2.

**Table 2:** Background characteristics

Characteristics	Group A *N* = 13	Group B *N* = 8	Group C *N* = 12
Gestational age (weeks); median	25.2	24.3	25.8
Range	24.0–26.4	24.0–26.3	23.6–26.6
IQR	0.8	0.7	1.0
Birth weight (g): median	650	730	868
Range	400–1130	365–820	480–990
IQR	360.0	305.0	231.3
Small for gestational age: *n* (%)	5 (38.5)	3 (37.5)	2 (16.7)
Gender			
Male: *n* (%)	9 (69.2)	4 (50)	6 (50)
Female: *n* (%)	4 (30.8)	4 (50)	6 (50)
Died: *n* (%)	4 (30.8)	4 (50)	0 (0)
Post natal age: median/IQR	51/24.5	30/34.3	
Antenatal steroids: *n* (%)	11 (84.6)	8 (100)	12 (100)
PIH: *n* (%)	3 (23.1)	2 (25)	2 (16.7)
Cesarean section: *n* (%)	9 (69.2)	5 (62.5)	7 (58.3)
Mechanical ventilation: *n* (%)	12 (92.3)	7 (87.5)	11 (91.7)
Surfactant treatment	10 (76.9)	7 (87.5)	11 (91.7)
Diuretics	11 (84.6)	4 (50)	11 (91.7)
Fluid restriction	9 (69.2)	3 (37.5)	8 (66.7)
PNA start PCM: median (days)	12.0	12.5	16.5
IQR	11.5	14.75	10.75
Paracetamol treatment	6.0	6.5	5.5
Days in total (median/IQR)	3	2.75	4
Surgical ligation: *n* (%)	5 (38.5)	6 (75.0)	12 (100)
PDA diameter before start PCM (mm): median/IQR	2.4/1.30	1.9/1.13	.4/0.83
PDA diameter after 3 days PCM (mm): median/IQR	1.9/0.90	2.1/1.13	2.1/1.08
PDA diameter after 7 days PCM (mm): median/IQR	1.8/1.28	2.0/0.40	2.6/1.60
PDA:LPA ratio before start PCM median/IQR	0.85/0.55	0.85/0.45	0.90/0.30
PDA:LPA ratio after 3 days PCM median/IQR	0.90/0.60	0.80/0.15	0.95/0.50
PDA:LPA ratio after 7 days PCM median/IQR	0.75/0.58	0.80/0.30	0.85/0.28
LA/Ao ratio before start PCM median/IQR	1.6/0.30	1.7/0.53	1.75/0.30
LA/Ao ratio after 3 days PCM median/IQR	1.64/0.60	1.7/0.70	1.8/0.55
LA/Ao ratio after 7 days PCM median/IQR	1.4/0.55	1.9/0.40	1.7/0.65
NT-proBNP (pmol/l)^a^: median	1097	2102	3078
IQR	3849.3	5832.0	4981.8

*PIH* pregnancy induced hypertension syndrome, *PNA* postnatal age, *PCM* paracetamol, *LPA* left pulmonary artery, *LA-Ao* left atrium-Aorta, *NT-proBNP* N-terminal pro-brain natriuretic peptide

^a^All NT-proBNP values were determined on day 3 PNA

Median duration of paracetamol treatment was 6 days (IQR 3); the median cumulative dose was 360 mg (IQR 180). Ductal closure or no hsPDA after treatment was achieved in six of the 33 patients (18 %). In total, 23 patients (76.7 %) needed surgical ligation for hsPDA with clinical symptoms. Findings in the three different groups (see Fig. 1) are detailed below.

Patients in group A (= primary contraindication for ibuprofen; *N* = 13, 39.4 %) received the first dose of paracetamol after a median of 12 (IQR 11.5) postnatal days and the median duration of the course was 6 (IQR 3) days. In six patients (46 %), the ductus arteriosus was closed or not hemodynamically significant after paracetamol and further treatment was not indicated. Two of the seven patients who did not respond to paracetamol treatment were successfully treated with ibuprofen (one and two courses, respectively) afterwards because the contraindication for ibuprofen had disappeared. Surgical ligation was performed in the other five (54 %).

Patients in group B (= incomplete ibuprofen; *N* = 8, 24.2 %) received the first dose of paracetamol after a median of 12.5 (IQR 14.75) postnatal days and the median duration of the course was 6.5 (IQR 2.75) days. Two patients died on the 24th and 34th postnatal day (i.e., sepsis with extension of bilateral intraventricular hemorrhage with venous infarction, gram-negative bacterial infection) after paracetamol treatment (started on day 8 and 13, respectively) before a decision could be made to treat the persistent PDA with surgical closure.

Paracetamol treatment was ineffective in the six remaining patients and all underwent surgical closure of the duct.

Patients in group C (= complete ibuprofen; *N* = 12, 36.4 %) received paracetamol for a median of 5.5 days (IQR 4) after two courses of ibuprofen. At the start of paracetamol treatment, their median postnatal age was 16.5 (IQR 10.75) days. Paracetamol treatment was ineffective in all patients, and consequently, they all underwent surgical ligation.

Both the surgical ligation rate and the mortality rate differed between the three groups. Surgical ligation was performed in 5/13 (38.5 %) patrients in group A; 6/8 (75 %) in group B; and 12/12 (100 %) in group C (Fisher exact test for surgical ligation *p* = 0.001). In total, eight patients died (Fisher exact test for death *p* = 0.025): 4/13 (31 %) in group A (median 51 days; IQR 24.5); 4/8 (50 %) in group B (median 30 days; IQR 34.3); and none in group C. Two of the four non-survivors in group B died from the consequences of NEC before surgical closure of the PDA could be performed.

**Fig. 2:**
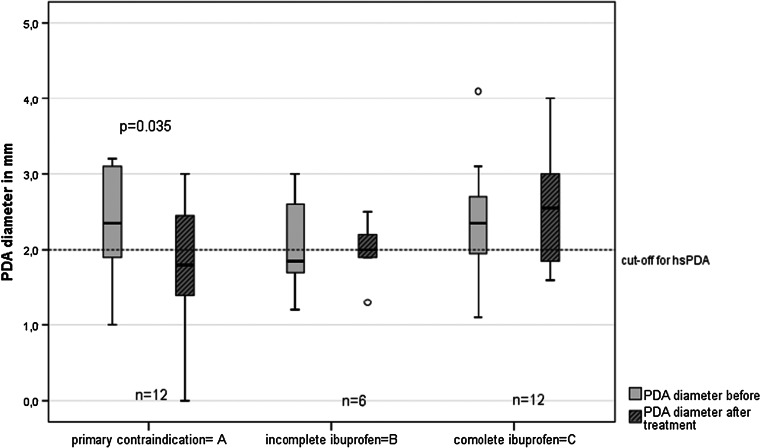
Change in ductus arteriosus diameter after 3 to maximum 7 days of intravenous PCM treatment for the three different groups (group A: primary contraindication for ibuprofen; group B: paracetamol after early stop of ibuprofen; and group C: paracetamol after complete ibuprofen treatment)

Cardiac ultrasound studies showed a statistically significant decrease in ductal diameter after paracetamol treatment only in group A, from median 2.4 to 1.8 mm after 7 days of treatment (*p* = 0.035, paired *t* test) (Fig. 2).

All pre- and posttreatment measurements of kidney function (urea and creatinine) and liver function (transaminases, conjugated bilirubin) were normal.

## Discussion

In this study, high-dose intravenous paracetamol for the treatment of hsPDA in VLBW infants was overall effective in only 18 %. Looking at the subgroups of patients, paracetamol treatment was completely ineffective after previous ibuprofen treatment failure. However, it was effective in 46 % of the newborns with primary contraindications for ibuprofen.

The variability in success rates of this paracetamol therapy for PDA closure is hard to explain. Other studies reported success rates of 0 [7] up to 100 % [12, 19]. Success rates in two RCTs comparing oral paracetamol versus oral ibuprofen were 81 [5] and 94 % [20], respectively, thus, much higher than in our study. Still, in all but one of those studies, the same dosing regimen of 60 mg/kg/24 h paracetamol was used. In the study by Tekgunduz et al. the dose was lowered to 30 mg/kg/day after a patient showed elevated transaminases [13]. The duration of paracetamol treatment in our study was 6.0 days compared to 4.1 days [29] and 3.9 days [19] in the studies with high success rates.

In previous studies, except in the two RCTs [5, 20], the indication for paracetamol was the same as in our study (treatment failure or contraindication for ibuprofen). The interpretation of different studies on this subject seems to be hindered by the lack of an international guideline on the definition of hsPDA and cutoff values for a small, medium, or large PDA (cardiac ultrasound measurements).

It is remarkable that Oncel et al. reported a 100 % success rate of PDA closure with intravenous paracetamol [19], the same administration route as in our study. Although unlikely, the route of administration could in part explain the variety in success rates; as long as data on bioavailability of the drug in the different routes and enterohepatic recirculation are lacking, we cannot tell which route of administration is superior. Still, ibuprofen studies also tend to find better results with oral administration [27]. Intravenous therapy probably leads to high peak plasma levels, but on the other hand, also to a relatively fast decrease in levels. Oral therapy might result in lower but more steady plasma levels. It can be hypothesized that PDA closure relies more on continuous prostaglandin inhibition than on intermittent high paracetamol or NSAIDs levels.

The relatively late start of paracetamol administration might be the most important explanation for our disappointing results compared to other studies. A second likely reason is the low gestational ages in our study. Other studies with better results [12, 19, 21, 29] included older infants with gestational ages >28 weeks. PDAs in this age group are generally less hemodynamically significant and tend to close spontaneously and respond better to pharmacological treatment.

Ibuprofen therapy failure was previously found to be 17 % in infants with gestational ages of 26–27 weeks versus 62 % in 23–25-week-old infants [6]. PDAs in more preterm born infants are probably less responsive to cyclooxygenase inhibitors due to higher expression of prostaglandin receptors [3]. Next to gestational age and postnatal age, clinical factors such as the amount of fluids given, incidence of infections or sepsis, type of respiratory support, and use of co-medication might be influential factors for PDA closure success in extreme preterm infants.

Third, selection bias may have occurred, in that, we included patients in whom ibuprofen therapy had failed prior to paracetamol treatment. As NSAIDs are more potent prostaglandin synthesis inhibitors than paracetamol [8], resulting in lower peripheral PGE2 levels as is shown in orthodontic studies [25], the a priori probability of success of paracetamol in this group of patients was already relatively low.

Pharmacokinetics and pharmacodynamics of paracetamol for PDA closure have been hardly studied. Consequently, the effective plasma level of paracetamol to achieve PDA closure is unknown. The currently used dosages are already much higher than recommended for analgesia, and might be unsafe. Increasing the dose to improve closure rates is unadvisable. In a study by Kessel et al., plasma levels of paracetamol (15 mg/kg/6 h by nasogastric tube) did not exceed the recommended plasma levels of 10–20 mg/l for pain and fever control [14]. Based on predictive modeling, plasma levels will accumulate with the 15 mg/kg/6 h regimen, reaching peaks of nearly 25 mg/kg after four doses [1]. In very preterm infants and murine studies, the effectiveness of paracetamol on PDA closure was suggested to depend on dosing, duration of treatment (>2 days course), and mode of administration [8].

Likewise, safety of paracetamol in very preterm infants (gestational age <28 weeks) has been little studied. Allegaert et al. showed no hemodynamic alterations during and following an intravenous loading dose of paracetamol and afebrile neonates maintained normothermia [2]. Paracetamol-induced hepatotoxicity is the most important concern; this is caused by the formation of a highly active metabolite *N*-acetyl-*p*-benzoquinone imine (NAPQ1) by the hepatic cytochrome P450-dependent CYP2E1 enzyme system [28]. The formation of NAPQ1 is suggested to be low due to the immaturity of the hepatic enzymes, although increased susceptibility to toxicity from supratherapeutic paracetamol is described in infants and younger children with fever [13]. CYP2E1 activity has not yet been quantified in neonates and the correlation between paracetamol concentration and increased NAPQ1 production is unknown.

Several limitations of this study should be addressed. First, this was an observational single center study with relatively few patients per group, without a matched control group to rule out spontaneous PDA closure. Firm statements are therefore difficult to make. Second, the echocardiosonographer and pediatric cardiologist were not blinded for the PDA treatment. Third, only patients were included in our study after ibuprofen failure or with a primary or secondary contraindication for ibuprofen; this selection bias might have contributed to our results.

## Conclusions

In view of the findings from this study, we do not recommend the use of intravenous paracetamol for hsPDA closure in VLBW infants after ibuprofen failure. Still, as we did not rule out effectiveness of paracetamol as early PDA treatment, it might be recommended when started in the first week of life. On the other hand, as long as data on long term safety are lacking, high dosages of paracetamol should be used with caution. Better designed PK/PD studies are needed to shed a light on safety aspects and the optimal dose-concentration-effect relationship. PK modeling of available data on PDA treatment with paracetamol in different gestational age groups can lead to different dosing recommendations per age group; a trial with these dose recommendations of paracetamol for PDA is also an option for future research.

## Abbreviations

hsPDA: Hemodynamically significant patent ductus arteriosus
IQR: Interquartile range
LA/Ao ratio: Left atrial to aortic root ratio
LPA: Left pulmonary artery
NEC: Necrotizing enterocolitis
NSAIDs: Non-steroidal anti-inflammatory drugs
PDA: Patent ductus arteriosus
VLBW: Very low birth weight
